# Evaluation of the Whole Proteome of *Achromobacter xylosoxidans* to Identify Vaccine Targets for mRNA and Peptides-Based Vaccine Designing Against the Emerging Respiratory and Lung Cancer-Causing Bacteria

**DOI:** 10.3389/fmed.2021.825876

**Published:** 2022-02-04

**Authors:** Taimoor Khan, Muhammad Abdullah, Tayyba Fatima Toor, Fahad N. Almajhdi, Muhammad Suleman, Arshad Iqbal, Liaqat Ali, Abbas Khan, Yasir Waheed, Dong-Qing Wei

**Affiliations:** ^1^Department of Bioinformatics and Biological Statistics, School of Life Sciences and Biotechnology, Shanghai Jiao Tong University, Shanghai, China; ^2^Amna Inayat Medical College, Lahore, Pakistan; ^3^Central Park Medical College, Lahore, Pakistan; ^4^Department of Botany and Microbiology, College of Sciences, King Saud University, Riyadh, Saudi Arabia; ^5^Centre for Biotechnology and Microbiology, University of Swat, Kanju, Pakistan; ^6^Division of Biology, Kansas State University, Manhattan, KS, United States; ^7^Foundation University Medical College, Foundation University Islamabad, Islamabad, Pakistan; ^8^State Key Laboratory of Microbial Metabolism, Joint Laboratory of International Laboratory of Metabolic and Developmental Sciences, Shanghai-Islamabad-Belgrade Joint Innovation Center on Antibacterial Resistances, Ministry of Education and School of Life Sciences and Biotechnology, Shanghai Jiao Tong University, Shanghai, China; ^9^Peng Cheng Laboratory, Shenzhen, China

**Keywords:** *Achromobacter xylosoxidans*, lungs cancer, vaccine targets, mRNA-based vaccines, immune simulation

## Abstract

*Achromobacter xylosoxidans* is a rod-shaped Gram-negative bacterium linked with causing several infections which mostly includes hematological malignancies. It has been recently reported to be associated with the development and progression of lung cancer and is an emerging respiratory disease-causing bacterium. The treatment of individuals infected with *A. xylosoxidans* bacteremia is difficult due to the fact that this pathogen has both intrinsic and acquired resistance mechanisms, typically resulting in a phenotype of multidrug resistance (MDR). Efforts are needed to design effective therapeutic strategies to curtail the emergence of this bacterium. Computational vaccine designing has proven its effectiveness, specificity, safety, and stability compared to conventional approaches of vaccine development. Therefore, the whole proteome of *A. xylosoxidans* was screened for the characterization of potential vaccine targets through subtractive proteomics pipeline for therapeutics design. Annotation of the whole proteome confirmed the three immunogenic vaccine targets, such as (E3HHR6), (E3HH04), and (E3HWA2), which were used to map the putative immune epitopes. The shortlisted epitopes, specific against Cytotoxic T Lymphocytes, Helper T-cell Lymphocytes, and linear B-Cell, were used to design the mRNA and multi-epitopes vaccine (MEVC). Initial validations confirmed the antigenic and non-allergenic properties of these constructs, followed by docking with the immune receptor, TLR-5, which resulted in robust interactions. The interaction pattern that followed in the docking complex included formation of 5 hydrogen bonds, 2 salt bridges, and 165 non-bonded contacts. This stronger binding affinity was also assessed through using the mmGBSA approach, showing a total of free binding energy of −34.64 kcal/mol. Further validations based on *in silico* cloning revealed a CAI score of 0.98 and an optimal percentage of GC contents (54.4%) indicated a putatively higher expression of the vaccine construct in *Escherichia coli*. Moreover, immune simulation revealed strong antibodies production upon the injection of the designed MEVC that resulted in the highest peaks of IgM+ IgG production (>3,500) between 10 and 15 days. In conclusion the current study provide basis for vaccine designing against the emerging *A. xylosoxidans*, which demands further experimental studies for *in vitro* and *in vivo* validations.

## Introduction

*Achromobacter xylosoxidans* is a motile, oxidase++, aerobic, and gram-negative rod-shaped bacterium extensively dispersed in the environment and reported to be associated with healthcare infection particularly the hematological malignancies (1). This bacterium has been reported to be associated with bacteremia in patients suffering from cystic fibrosis (2). The emerging bacterium is also associated with lung disease severity in children and lung inflammation in patients (3, 4). The bacterium mainly colonizes in the patient's airway and follows a complex mechanism of evolutionary dynamics related with host/pathogen interactions (5). It was originally isolated from patients with otitis media, where associated complications including pharyngitis, pneumonia, peritonitis, and urinary tract infections (6). Moreover, *A. xylosoxidans* has been recently reported to be associated with the development and progression of lung cancer. It is also reported as an emerging respiratory disease-causing bacterium (7). Infection in the lungs, with *A. xylosoxidans*, has been linked to a variety of health complications, notably IgM deficiency and acute myelogenous leukemia (AML) (8, 9).

The treatment of individuals infected with *A. xylosoxidans* bacteremia is difficult due to the fact that this pathogen has both intrinsic and acquired resistance mechanisms, typically resulting in a phenotype of multidrug resistance (MDR) (10). It is resistant to all the aminoglycosides and rifampin, as well as trimethoprim-sulfamethoxazole, ciprofloxacin, and other quinolones, with varying resistance to trimethoprim-sulfamethoxazole, ciprofloxacin, and other quinolones. Carbapenems and antipseudomonal penicillin are typically effective against most isolates (11–13). However, there is no specific treatment for this multi-disease-causing bacterium. Efforts are needed to design effective therapeutic strategies to curtail the emergence of this bacterium. Designing small molecule inhibitors is a time taking process, however, vaccination has been used to prevent and to cure a range of microbial infections, with the goal of generating adaptive immune responses by delivering antigenic components to the immune system (14). Classical vaccines, like inactivated pathogens, subunit vaccines, and live attenuated provide long-lasting protection, but they limit quick and large-scale production possibilities (15). The use of computational tools has significantly reduced the cost and time of developing peptides-based therapeutics. Computational vaccine designing has proven its effectiveness, specificity, safety, and stability compared to the conventional approaches of vaccine development (14, 16–19). Immunoinformatic strategy has been adopted for designing vaccine against a number of pathogens including severe acute respiratory syndrome coronavirus 2 (SARS-CoV-2), Mayaro virus, human norovirus, and Shigella spp (14, 16–18, 20).

Computational methods are of great interest in exploring biological mechanisms and designing therapeutics against the emerging pathogens (21, 22). This also involves the application of computational vaccinology approaches to design vaccine therapeutics. In this scientific study, screening of the whole proteome for vaccine targets prioritization, followed by mapping of cytotoxic T-cell Epitopes, helper T-cell epitopes, and linear B cell epitopes, extracted from the antigenic proteins of *A. xylosoxidans*, was performed. The shortlisted proteins selected from the extracellular membrane are reported to be involved in attachment, pathogenesis, replication, and disease severity. Antigenicity, immunogenicity, and allergenicity of the selected epitopes were predicted to design a potent peptide vaccine composed of potential antigenic messenger RNA (mRNA) and peptides-based multi-epitopes vaccines. The current findings will aid the development of potential peptides-based vaccine candidate. This research could pave the way for the development of a dynamic and efficient multi epitopes-based vaccine that contain a unique mix of numerous *A. xylosoxidans*-derived antigenic peptides with different roles during the *A. xylosoxidans* infection. Overall, this may also advance the therapeutics research to combat the emerging pathogen.

## Methodology

### Proteome Subtraction

The total proteome (UniProt ID: UP000006876) of *A. xylosoxidans* was accessed and the chromosome region (Genome accession: CP002287) coding 6,445 proteins was downloaded from UniProtKB (https://www.uniprot.org/) (23). The whole proteome sequences were then subjected to subtractive proteomics approach for the identification of target proteins to design putative vaccine candidates against the target proteins of *A. xylosoxidans*. Firstly, each protein was screened on the basis of a subcellular localization with the utility of an online server, CELLO (http://cello.life.nctu.edu.tw/) (24). The predictions of this integrated server are based on a two-level support vector machine (SVM) system to determine the protein localization. Secondly, to exclude pathogen and human host (Homo sapiens, ID: 9606) homologous proteins; BLASTp tool with default parameters (e-value: 10-5) was utilized. This was performed to remove homologous proteins and to further process the non-homologous proteins in vaccine target prioritization (25). Furthermore, the Cluster Data Base with High Tolerance (CD-HIT) suite (http://weizhongli-lab.org/cd-hit/) (26) was utilized to exclude overlapping protein sequences in the whole proteome. This analysis based on an improved clustering algorithm was performed to remove duplicated proteins with a cut-off value of 0.8 representing 80% identity (27). Moreover, shortlisting of immunogenic target proteins was performed with the utility of two web servers, VaxiJen (http://www.ddg-pharmfac.net/vaxijen/VaxiJen/VaxiJen.html) (28) and Algpred2 (https://webs.iiitd.edu.in/raghava/algpred2/) (29). The analysis was deployed to characterize the pathogenic proteins on the basis of antigenicity and allergenicity status. Finally, the shortlisted three target proteins were analyzed during additional investigations.

### Epitopes Prioritization in *A. xylosoxidans* Proteome

The process of putative epitopes screening was initiated with the utility of NetCTL 1.2 (http://www.cbs.dtu.dk/services/NetCTL/) server (30) to predict Cytotoxic T-lymphocytes. The method, trained on 886 known MHC class I ligands and 12 super-types, helps to predict the CTL epitopes based on TAP (Transport Associated with Antigen Processing). This was followed by prediction of HTL epitopes using the IEDB online server (http://www.iedb.org/) by using seven reference alleles set of human HLAs (31). The server predicts and characterizes the HTL epitopes based on binding affinity by depicting percentile ranks, whereas an epitope with a lower percentile rank indicates a higher binding affinity. Furthermore, to investigate the interferon-gamma producing helper T lymphocyte (HTL), epitopes were screened using an IFN- epitope (http://crdd.osdd.net/raghava/ifnepitope/) server (32). The analysis was performed with the utility of server integrated algorithms, which classifies the HTL epitopes based on SVM scores to differentiate between IFN-positive (inducing) and IFN-negative (non-inducing) epitopes (32). This was proceeded with prediction of linear B-cell epitopes with the utility of ABCPred online server (https://webs.iiitd.edu.in/raghava/abcpred/) (33). These investigations are based on comparative analysis with experimentally proved continuous B cell epitopes with higher accuracy (34). Additionally, the final shortlisting of these epitopes for inclusion in vaccine designs were preceded by antigenic and allergenic potential evaluation. The highly antigenic and non-allergenic epitopes were included in the process of *in silico* vaccine designing.

### Proteome-Wide mRNA-Based Vaccine Design

The selected highly antigenic epitopes that are predicted for each target protein were then utilized in the design of an mRNA-based vaccine against *A. xylosoxidans*. Herein, 2 T-cell epitopes from each target protein were selected and a total of 6 CTL epitopes were included in the final whole proteome specific mRNA-based vaccine design (35). Similarly, a total of 6 HTL and 6 B-cell epitopes were included in the final mRNA vaccine candidate. Furthermore, an addition of 5′m7G Cap, 5′ untranslated region (UTR), Kozak sequence, signal peptide, and linked epitopes with suitable linkers (AAY, PMGLP, and GGGGS) was consecutively performed (36). A stop codon was then added before the 3′ UTR region and was followed by the poly A Tail. The same assembly of epitopes was used in the design of multi-epitopes-based and proteome-wide peptides-based vaccine design.

### Proteome-Wide Peptides-Based Vaccine Assembly and Three-Dimensional Structure Modeling

The final multi-epitopes-based putative vaccine candidate against *A. xylosoxidans* was designed based on the same assembly of the highly antigenic and non-allergenic 6 CTL, 6 HTL, and 6 B-cell epitopes. The three different classes of epitopes were added with a suitable adjuvant and were linked together by using different linkers (EAAK, sAAY, GPGPG, and KK, respectively) (16, 22, 37). This addition of linkers and adjuvant is important to retain the independent immunogenic activity of epitopes and to prevent differentiation after inclusion in the vaccine construct (38). After finalizing the assembly of the whole proteome-based vaccine construct, Robetta online server (http://robetta.bakerlab.org) (39) was utilized for modeling of the 3D structure. The server is capable of modeling multi-chain complexes by using the integrated RoseTTAFold or comparative modeling approach to model (3D) the input amino acid sequences.

### Validation of the Peptides-Based Vaccine Design

To validate the accuracy of the 3D structure design, several protein structure validation servers were employed. This included the utility of proSA-web (https://prosa.services.came.sbg.ac.at/prosa.php) (40) server to predict the Z-score of the 3D model. The deviations in Z-score from the normal range are indicative of errors in the tertiary structure of modeled proteins. Additionally, ERRAT online server (https://www.doe-mbi.ucla.edu/errat/) was utilized to predict the quality factor based on the atomic bonding contacts. Finally, the available online server, PROCHECK (https://servicesn.mbi.ucla.edu/PROCHECK/) (41), was used to assess the stereo-chemical properties and to predict the overall structure geometry through Ramachandran Plot analysis (42, 43). Finally, the different physiochemical properties of the peptides-based vaccine construct were evaluated by using the ProtParam online tool (http://web.expasy.org/protparam/) (44). This was performed to depict several topographies of the constructed vaccine including molecular weight, theoretical PI, instability index, and other related properties.

### Docking and Interaction Analysis of the Vaccine

To identify the interacting patterns, molecular docking of the proposed vaccine with human TLR-5 (Toll-like receptor-5) (45) was performed using the HawkDock server (http://cadd.zju.edu.cn/hawkdock/). This server, by deploying a hybrid docking method, efficiently provides information about the binding interactions of the complex (42). Additionally, the server also offers an mm-GBSA analysis for binding free energy calculations. The structure of TLR-5, utilized in the docking complex, was retrieved from RCSB using the Accession ID: 3j0a. The structure of TLR-5 was visualized and was prepared in PyMOL before docking with the proposed vaccine.

### *In silico* Cloning of the MEVC

The *in silico* cloning analysis were initiated with the acquisition of reverse-translated optimized DNA sequence for the peptides-based vaccine construct. This was performed with the utility of Java codon adaptation tool (JCat tool) to ensure the production of the multi-epitope subunit vaccine in an appropriate expression host (43). After selecting appropriate host (E. coli strain K-12) for the proposed vaccine, the GC content and the CAI score of the optimized DNA sequence were also determined. Furthermore, the optimized DNA sequence, after choosing two restriction enzyme sites (XhoI and EcoRI), was inserted in the pET-28a (+) expression vector to obtain the cloned plasmid by using the Snapgene software.

### Immune Simulation

The validation of potential immunogenic response induced by the proposed peptides-based vaccine against the *A. xylosoxidans* was also performed. This was achieved with the utility of immune simulation approach by using C-ImmSim (http://150.146.2.1/C-IMMSIM/index.php) (46). The server is capable of depicting putative immune responses against the desired antigenic protein constructs. Several machine learning methods, with scoring matrix PSSM-based systems, are deployed to forecast antibody responses. This evaluation includes counts of antibodies, cytokines, and interferons produced against an injected antigen (47). The method is widely deployed to depict the experimental feasibility of computationally designed vaccines against the target organisms.

### Molecular Dynamics Simulation

Molecular dynamics (MD) simulation of the vaccine-TLR complex was performed to check the stability of the complex using AMBER20 simulation package for 20ns (48, 49). The parameters were used as previously used by Abbas et al. (50, 51). For stability and residual flexibility estimation, CPPTRAJ and PTRAJ modules were used (52).

## Results

### Cellular Localization and Immunogenic Potential Based Protein Targets Prioritization

**Figure 1:**
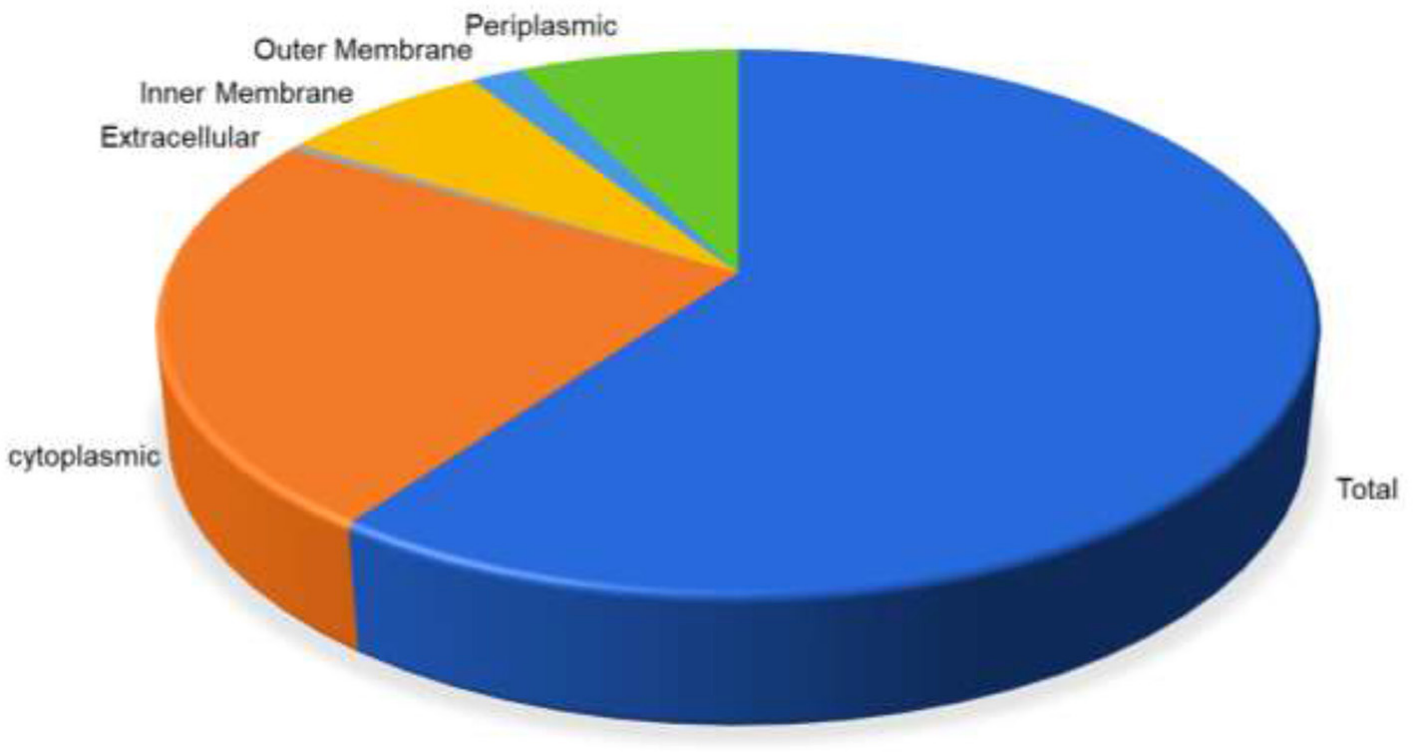
Distribution of proteins based on the location inside the cell. Periplasmic (green) are 7.32%, outer membrane (light blue) 1.72%, inner membrane (yellow) 6.94%, extracellular (gray) 0.45%, while cytoplasmic proteins are 23.66%, respectively.

The whole proteome sequence of pathogenic *A. xylosoxidans* was subjected to the mining of the therapeutic targets. This was performed to shortlist the novel protein targets for computational vaccine designs. The approach is widely utilized to identify the genome-wide therapeutic targets in several diseases (53). Herein, subtractive proteomics pipeline was followed to shortlist putative vaccine targets against *A. xylosoxidans*. This was initiated with the screening of subcellular localization for the whole proteome, which contains a total of 6,445 proteins. The screening was performed through the online available protein localization tool “CELLO” (http://cello.life.nctu.edu.tw/). This resulted in a proteome-wide localization for each individual protein classified as periplasmic, inner membrane, and outer-membrane proteins, as shown in Figure 1. This helped in the shortlisting of 185 outer membrane proteins as the prioritized vaccine targets which are processed in further analysis (54, 55). These proteins were then screened for removal of human homologous proteins through using BLASTp tool, followed by paralogs screening using CD-HIT. Furthermore, the shortlisted proteins were also screened for antigenicity and allergenicity profiles, to remove non-antigenic and allergenic proteins. This characterization resulted in the selection of the highly immunogenic three target proteins with antigenicity score of >0.8. These highly antigenic targets included an outer membrane transport protein, an uncharacterized protein, and a Filamentous hemagglutinin family N-terminal domain protein, as shown in Table 1. Same protein targets were then analyzed to screen putative immune epitopes (T-Cell epitopes, HTL epitopes, and B-Cell epitopes) for inclusion in the final vaccine designs.

**Table 1:** The prioritized outer-membrane target proteins with antigenicity scores of ≥0.8.

**UniProt accession**	**Target outer membrane protein**	**Antigenicity scores**
E3HHR6	Uncharacterized protein	0.80
E3HH04	Outer membrane transport protein OMPP1/FadL/TodX family protein	0.85
E3HWA2	Filamentous hemagglutinin family N-terminal domain protein 1	0.88

### Prioritization of Highly Immunogenic Epitopes for Each Target Protein

The identification of immune epitopes through advanced computational approaches ultimately aids in designing the highly antigenic vaccine designs against the pathogenic organisms. The selection of the target proteins was followed by screening of putative immunogenic epitopes (CTL, HTL, and B-cell). The analysis was initiated with identification of CTL epitopes in each target protein sequence. These epitopes are the essential factors involved in the recognition through MHC molecules for production of immune response. Similarly, the antigenic HTL and B-cell epitopes recognition by the host immune system are also vital in mediating adaptive immunity. Next, the shortlisted three proteins of *A. xylosoxidans* were also screened for identification of HTL epitopes with an IFN induction potential. This was followed by the identification of B-cell epitopes for each target protein. During the screening of T-Cell Epitopes, a total of 9, 10, and 70 MHC-I binders were identified for each target protein, respectively; whereas the number of identified HTL epitopes were 1,953, 2,982, and 1,070 while B-Cell epitopes were 31, 47, and 540, respectively, characterized for each of the three target proteins. Additionally, the epitopes selected among the identified immune epitopes was subjected to further analysis for antigenicity and allergenicity potential before the inclusion in the final vaccine designs. The final shortlisted epitopes after immunogenic potential evaluation based on high antigenicity scores are given in Table 2. All the shortlisted epitopes (CTL, HTL, and B cell) from Table 2 were included in the MEVC designs against *A. xylosoxidans*.

**Table 2:** The shortlisted immune epitopes with antigenicity and allergenicity status evaluated for each target protein.

**Target protein accession ID**	**Type of epitopes**	**Total shortlisted epitopes**	**Epitope sequences**	**Antigenicity scores**	**Antigenicity status**	**Allergenicity**
E3HHR6	CTL	2	SVDVNTLVY TLGSAIWFY	1.2 1.1	Antigenic	Non-allergenic
E3HHR6	HTL	2	VGTNIIAGYMAGLRS FAWEPFVVAPVGRYD	0.5 0.7	Antigenic	Non-allergenic
E3HHR6	B-cell	2	EVEIYGRNTDWHGQTL PGMAGDHQTGIGDVTL	0.7 1.1	Antigenic	Non-allergenic
E3HH04	CTL	2	TTLDVGYTY NASGLGNAY	0.4 0.9	Antigenic	Non-allergenic
E3HH04	HTL	2	YDSNAHILGIQLSSR ALGANYKFAPNWKWK	0.9 2	Antigenic	Non-allergenic
E3HH04	B-cell	2	TGLGGSRPTGGNGGDA SWTGWSSIPNLKIRNS	2.3 0.5	Antigenic	Non-allergenic
E3HWA2	CTL	2	ETSPITPTY QSDASMTLQ	0.4 1.1	Antigenic	Non-allergenic
E3HWA2	HTL	2	AGALRSNQGNRIEAG GSAAQLQSSKGMTLS	1 1.1	Antigenic	Non-allergenic
E3HWA2	B-cell	2	PGTIEVRSDKDSGRDS GGSIVHGGTSLAGKDL	1.5 0.6	Antigenic	Non-allergenic

### Designing mRNA- and Peptides-Based MEVC

**Figure 2:**
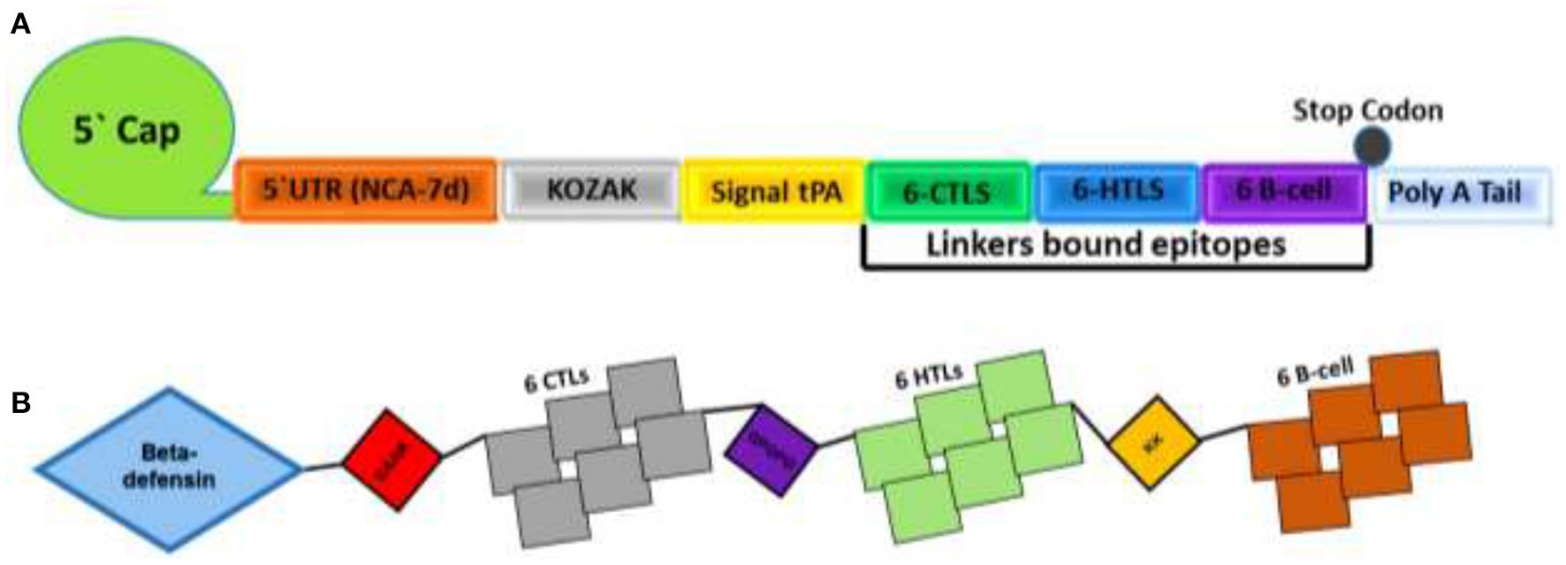
Topological representation of the designed vaccines. **(A)** Final mRNA vaccine construct with an organized order of elements from left (N-terminal) to right (C-terminal). Each structural element, such as 5′ cap, 5′ UTR, Kozak Sequence, Signal protein, CTL epitopes, HTL epitopes, B-cell epitopes, 3′ UTR, and a Poly A tail, is represented by a different color scheme. **(B)** shows the final peptides-based vaccine construct with the adjuvant attached at N terminal and different epitopes are linked by EAAK, AAY, GPGPG and KK linkers.

The proteome-wide identification of putative targets and the mapping of vital epitopes was followed by mRNA and by peptides-based vaccine designing. The highly antigenic epitopes, joined together through different linkers, were then used in the full-length mRNA- and peptides-based MEVC design shown in Table 3. The mRNA-based vaccine against *A. xylosoxidans* was designed by targeting the three highly antigenic target proteins. The assembly of final mRNA vaccine construct comprised of a total of 18 immune epitopes (6 CTL epitopes, 6 HTL epitopes, and 6 linear B-cell epitopes). This was initiated with the N terminal 5'm7G cap followed by NCA-7d (5' UTR), Kozak sequence, and a signal peptide tPA (tissue Plasminogen Activator). The assembly of mRNA vaccine also includes the addition of a stop codon, S27a+R3U (3' UTR) sequence, and a Poly (A) tail (120 nucleotides long). The graphical representation and arrangement of the mRNA vaccine design is shown in Figure 2A. The peptides-based whole proteome-wide vaccine construct against *A. xylosoxidans* of length 365 amino acids was also evaluated for different physiochemical properties, along with antigenic and allergenic potential to ensure its application in further experimental designs (Figure 2B).

**Table 3:** The full-length peptides-based MEVC design with explored physiochemical properties.

**Vaccine type**	**Proteome wide peptides based MEVC Construct**	**Number of amino acids**	**Molecular weight (kd)**	**Theoretical pI**	**Aliphatic index**	**Hydropathicity (GRAVY)**	**Antigenicity score**	**Antigenicity status**	**Allergenicity**
mRNA based vaccine	SVDVNTLVYAAYTLGSAIWFYAAYTTLDVGYTYAAYNASGLGNAYAAYETSPITPTYAAYQSDASMTLQGPGPGVGTNIIAGYMAGLRSGPGPGFAWEPFVVAPVGRYDGPGPGYDSNAHILGIQLSSRGPGPGALGANYKFAPNWKWKGPGPGAGALRSNQGNRIEAGGPGPGGSAAQLQSSKGMTLSKKEVEIYGRNTDWHGQTLKKPGMAGDHQTGIGDVTLKKTGLGGSRPTGGNGGDAKKSWTGWSSIPNLKIRNSKKPGTIEVRSDKDSGRDSKKGGSIVHGGTSLAGKDL	297	30 kd	9.54	62.53	−0.465	1	Antigen	Non-allergen
Peptides based vaccine	MRVLYLLFSFLFIFLMPLPGVFGGIGDPVTCLKSGAICHPVFCPRRYKQIGTCGLPGTKCCKKPEAAKSVDVNTLVYAAYTLGSAIWFYAAYTTLDVGYTYAAYNASGLGNAYAAYETSPITPTYAAYQSDASMTLQGPGPGVGTNIIAGYMAGLRSGPGPGFAWEPFVVAPVGRYDGPGPGYDSNAHILGIQLSSRGPGPGALGANYKFAPNWKWKGPGPGAGALRSNQGNRIEAGGPGPGGSAAQLQSSKGMTLSKKEVEIYGRNTDWHGQTLKKPGMAGDHQTGIGDVTLKKTGLGGSRPTGGNGGDAKKSWTGWSSIPNLKIRNSKKPGTIEVRSDKDSGRDSKKGGSIVHGGTSLAGKDL	365	37 kd	9.55	67.70	−0.283	1	Antigen	Non-allergen

#### Structural Modeling and Validation of MEVC

**Figure 3:**
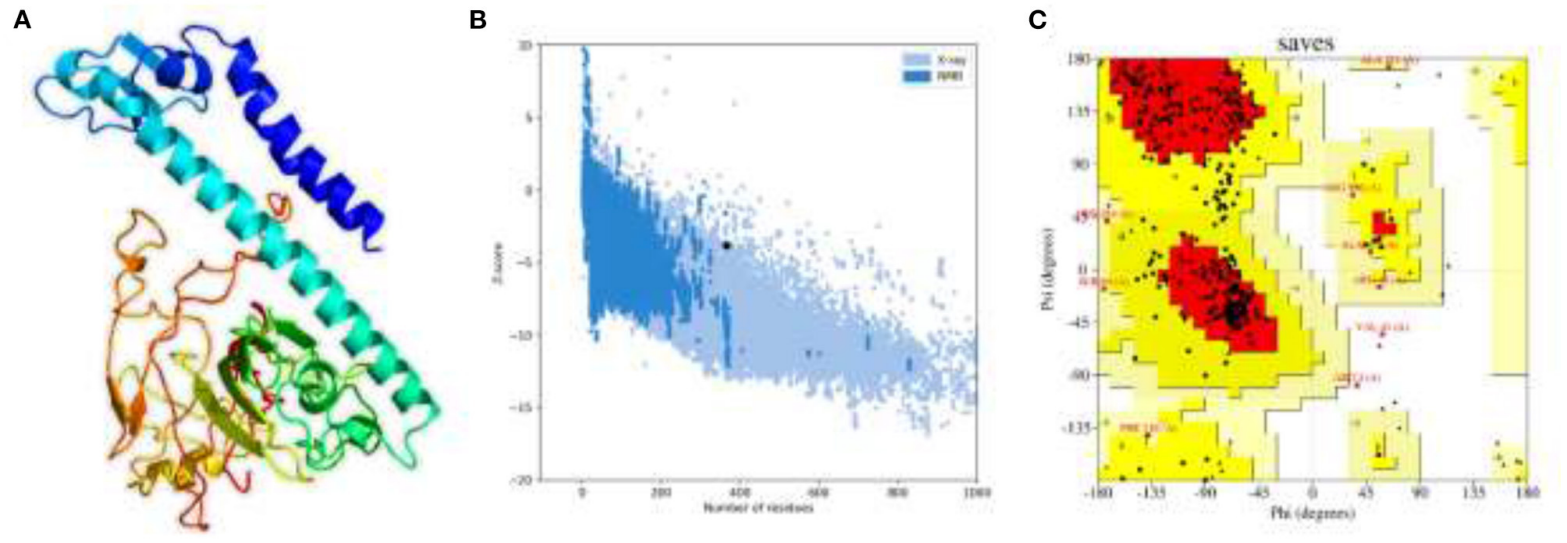
The 3D structural model and validation of the final multi-epitopes-based vaccine design against *A. xylosoxidans*. **(A)** The Robetta generated 3D model of the vaccine construct, **(B)** the ProSA-web that generated a Z-score graph, and **(C)** the PROCHECK-generated Ramachandran-plot for validation of the structure.

Structural vaccinology approaches, by using computationally predicted epitopes, have been already deployed in several experimental models (56, 57). Here, in the whole proteome-wide, final peptides-based MEVC construct of length 365 amino acids, involving 18 immune epitopes joined through linkers and N-terminal adjuvant, was constructed. Next, the 3D structure of the final vaccine construct was generated with the utility of Robetta server as shown in Figure 3A. The best 3D predicted model was visualized using PyMOL software and was then validated through two different servers to finalize the most accurate model. This model validation was performed with the use of ProSA-web and PROCHECK servers. The ProSA-web analysis revealed a Z-score of−3.8 (Figure 3B) and the ERRAT predicted high quality scores that indicated the validity of the predicted 3D structure. Moreover, PROCHECK resulted in the Ramachandran-plot that showed 72.5% of the residues in the most favored regions and only 0.7% in the dis-allowed region (Figure 3C).

### Docking Complex Analysis of MEVC-TLR5 and Interaction Analysis

**Figure 4:**
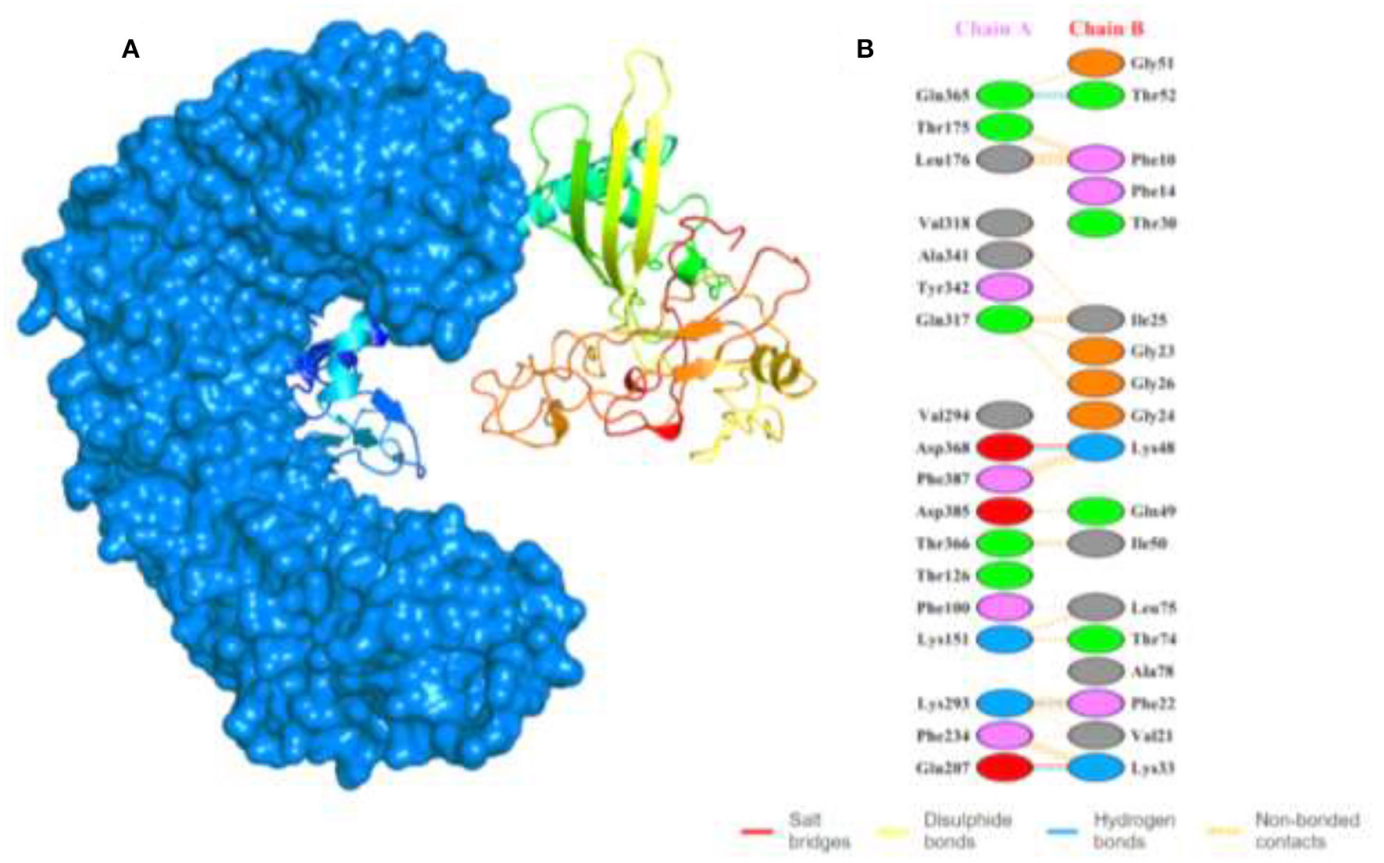
Shows the docking complex of the vaccine structure and human TLR-5. **(A)** The docking complex with human TLR-5 structure represented in surface view (marine blue color), while the multi-colored attached structure represents the designed MEVC. **(B)** The binding patterns formed in the docking complex between the two interacting partners.

To evaluate the MEVC-TLR5 interactions, we utilized the HawkDock server to generate several docking models with docking complex scores. The best docking complex (Figure 4A) with lowest acquired energy scores (−5673.28) were then subjected to interaction analysis by utilizing PDBsum. This was performed to calculate the number of different binding interactions including hydrogen bonds, salt bridges, and non-bonding contacts in the docking complex as shown in Figure 4B. The interaction pattern, followed in the docking complex, included formation of 5 hydrogen bonds, 2 salt bridges, and 165 non-bonded contacts. Furthermore, the MM-GBSA analysis was also performed to reveal the total and individual binding free energies involved in the regulation of the formation of the docking complex. The energies are as follows: Van der Waals energy (−103.99 kcal/mol), Electrostatic energy (−1833.88 kcal/mol), Gibbs free energy (1915.62 kcal/mol), Surface Area (−12.39 kcal/mol), and the total binding energy −34.64 kcal/mol, which shows that robust binding of the vaccine to the TLR-5.

### *In silico* Cloning Design of WP-MEVC

**Figure 5:**
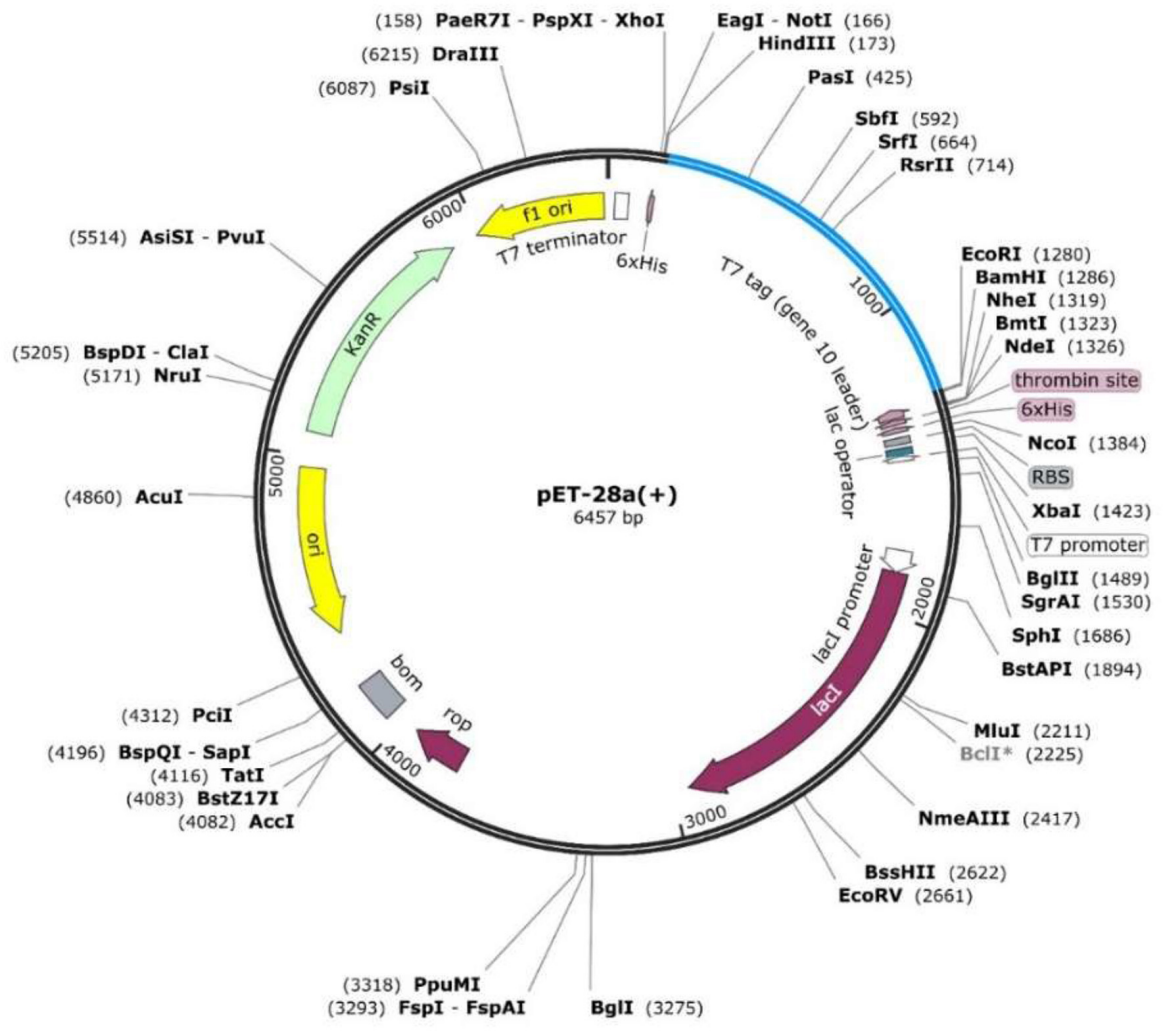
The designed plasmid map for the proposed vaccine construct by using restriction enzymes (XhoI and ECOR1) in the pet28a (+) vector.

The constructed peptides-based vaccine design was also subjected to the acquisition of an improved DNA sequence for the expression in the *E. coli* strain K-12 with the utility of Jcat server. The optimized nucleotide sequences with a calculated higher CAI score of 0.98 and an optimal percentage of GC contents (54.4%) indicated a putatively higher expression of the vaccine construct in *E. coli*. This was followed by the selection of the restriction enzymes (XhoI and ECORI) used in the cloning of the optimized DNA sequence in pET28a (+) vector. The insertion of the desired vaccine sequence, followed by designing of pET28a (+) plasmid, was performed in Snapgene (Figure 5).

### Immune Simulation of the Proposed Vaccine

**Figure 6:**
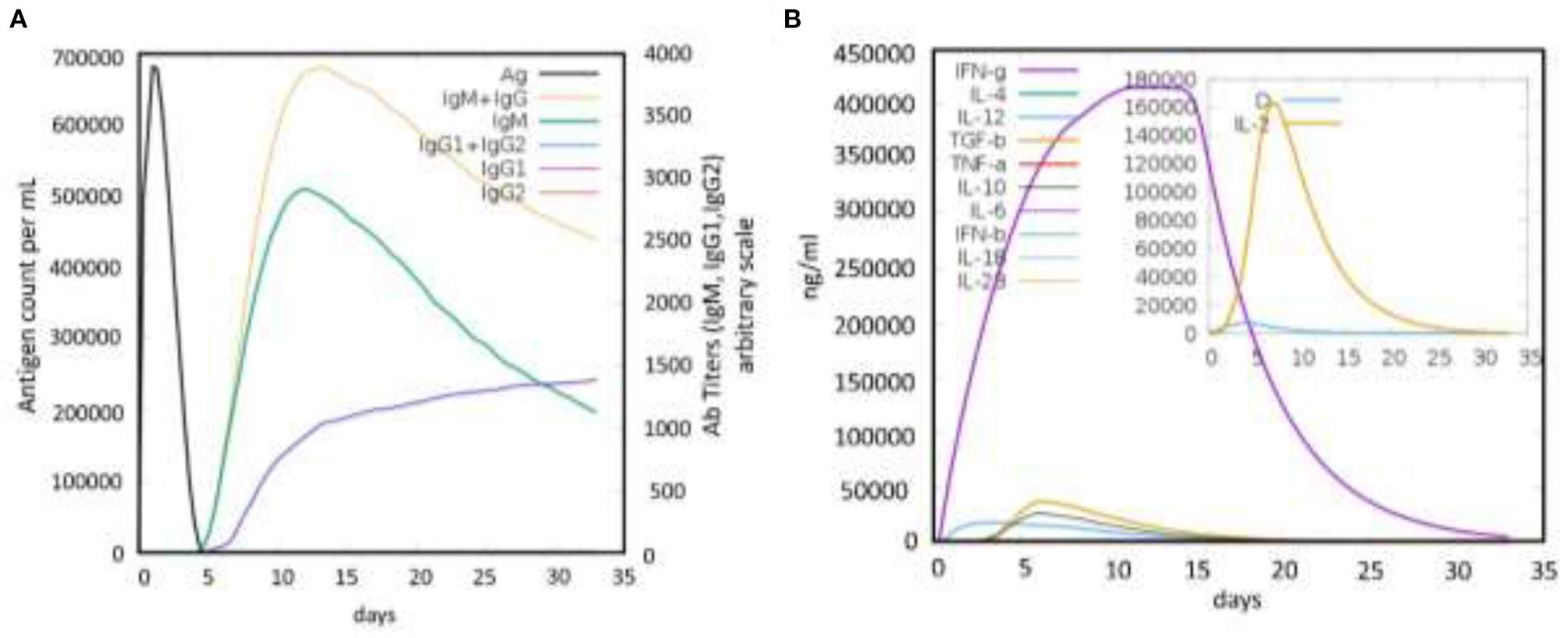
The immune simulation graphs showing immune response produced against the antigenic peptides-based vaccine design. **(A)** The graph showing antigen count/ml/day plotted against Ab titers. **(B)** The production of cytokines and interleukins against the whole proteome-wide peptides-based vaccine design.

Finally, the immune response induction potential of the designed vaccine was also evaluated for the highly antigenic MEVC construct during the *in silico* immune simulation. The analysis revealed a strong induction of antibodies production against the vaccine construct as an antigen. This analysis showed a higher antigen counts of >600,000 between days 1-2 and were completely neutralized until the 5th day. This was followed with an increasing trend of antibodies produced against the antigenic vaccine, achieving the highest peaks (>3,500) of IgM+ IgG production between 10 and 15 days (Figure 6A). Similarly, higher immunoglobulins (IgM, IgG1, and IgG2), specific antibody titers, were also observed during the same time period. These results reflected the putative immunogenic potential of the designed MEVC to trigger an enhanced immune response against *A. xylosoxidans*. Additionally, the peptides-based vaccine design was also evaluated for production of different Interleukins (IL), Interferon (IFN), transforming growth factors (TGF), and transforming necrosis factor (TNF). The highest among these factors, produced against the antigenic vaccine, was IFN-g with the highest peak (>400,000) observed during 10 to 15 days. The concentrations of different cytokines and interleukins produced are presented in Figure 6B. However, an advanced experimental analysis of the designed vaccine candidate and its potential to produce adaptive immunity against human pathogenic *A. xylosoxidans* may be clarified through further *in vitro* and *in vivo* demonstrations.

### MD Simulation

**Figure 7:**
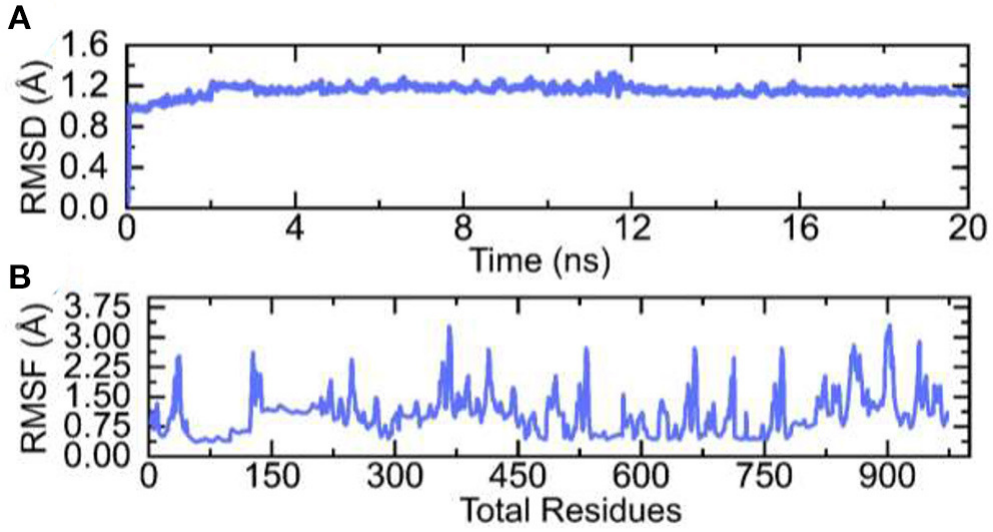
RMSD and RMSF graphs of the vaccine-TLR complex over the simulation time. **(A)** RMSD and **(B)** RMSF of the complex.

Molecular dynamics (MD) simulation of the vaccine-TLR complex was performed to check the stability and flexibility of the complex. The stability of the complex was calculated as room mean square deviation (RMSD), which revealed the stable behavior of the complex over the simulation time. The RMSD graph of the complex is given in Figure 7A. On the hand the residual flexibility was calculated as room mean square fluctuation (RMSF), which revealed different flexibility index of each residue (Figure 7B). Overall, the flexibility level is well acceptable and demonstrates the favorable dynamic behavior.

## Conclusions

In conclusion, the whole proteome of *A. xylosoxidans* was studied to shortlist the three putative vaccine targets. This was followed by the prediction of different immune epitopes (CTL, HTL, and B-cell) for each of the target protein. The highly immunogenic peptides were then topographically arranged to design a proteome-wide mRNA and MEVC. The final vaccine construct was then subjected to a 3D modeling and structural validation. The evaluations of the vaccine structure and exploration of physiochemical features also reflected the ability of a potential immunization. Moreover, strong interaction patterns (more hydrogen bonds), through molecular docking and experimental feasibility, were confirmed through *in silico* cloning and immune simulations. The study provides new insights into target specific immune epitopes identification, and its utility in mRNA and peptides-based vaccine designs. This also suggests further experimental validation of the proposed vaccine designs. However, the experimental processing may face challenges including protein expression solubility during synthesis. Still, the vaccine designs may serve as pre-validated therapeutic option with a potential utility against *A. xylosoxidans*.

## Data Availability Statement

The datasets presented in this study can be found in online repositories. The names of the repository/repositories and accession number(s) can be found in the article/supplementary material.

## Author Contributions

TK, FA, TT, MS, and AK: conceptualization. MA: data curation. TK, FA, MS, and AK: formal analysis. TK, FA, TT, and AI: investigation. MA and TT: methodology. AI and D-QW: resources. MA, MS, AI, and LA: software. LA and D-QW: supervision. MS, LA, AK, and YW: validation. LA: visualization. AK, YW, and D-QW: writing—original draft. YW and D-QW: writing—review and editing.

## Funding

FA is funded from Researchers Supporting Project of King Saud University, Riyadh, Saudi Arabia (No. RSP-2021/198).

## Conflict of Interest

The authors declare that the research was conducted in the absence of any commercial or financial relationships that could be construed as a potential conflict of interest. The reviewer MMSB declared a shared affiliation with one of the author, FA, to the handling editor at the time of review.

## Publisher's Note

All claims expressed in this article are solely those of the authors and do not necessarily represent those of their affiliated organizations, or those of the publisher, the editors and the reviewers. Any product that may be evaluated in this article, or claim that may be made by its manufacturer, is not guaranteed or endorsed by the publisher.
